# Cilia proteins are biomarkers of altered flow in the vasculature

**DOI:** 10.1172/jci.insight.151813

**Published:** 2022-03-22

**Authors:** Ankan Gupta, Karthikeyan Thirugnanam, Madhan Thamilarasan, Ashraf M. Mohieldin, Hadeel T. Zedan, Shubhangi Prabhudesai, Meghan R. Griffin, Andrew D. Spearman, Amy Pan, Sean P. Palecek, Huseyin C. Yalcin, Surya M. Nauli, Kevin R. Rarick, Rahima Zennadi, Ramani Ramchandran

**Affiliations:** 1 Department of Pediatrics and; 2 Division of Neonatology, Department of Pediatrics, and Developmental Vascular Biology Program, Children’s Research Institute (CRI), Medical College of Wisconsin, Milwaukee, Wisconsin, USA.; 3 Division of Hematology, Department of Medicine, School of Medicine, Duke University, Durham, North Carolina, USA.; 4 Department of Pharmaceutical Sciences, School of Pharmacy, Chapman University, Irvine, California, USA.; 5 Biomedical Research Center, Qatar University, Doha, Qatar.; 6 Division of Cardiology and; 7 Division of Quantitative Health Sciences, Department of Pediatrics, CRI, Medical College of Wisconsin, Milwaukee, Wisconsin, USA.; 8 Department of Chemical and Biological Engineering, College of Engineering, University of Wisconsin-Madison, Madison, Wisconsin.; 9 Division of Critical Care, Department of Pediatrics, CRI, Medical College of Wisconsin, Milwaukee, Wisconsin, USA.

**Keywords:** Vascular Biology, Endothelial cells, Molecular diagnosis

## Abstract

Cilia, microtubule-based organelles that project from the apical luminal surface of endothelial cells (ECs), are widely regarded as low-flow sensors. Previous reports suggest that upon high shear stress, cilia on the EC surface are lost, and more recent evidence suggests that deciliation—the physical removal of cilia from the cell surface—is a predominant mechanism for cilia loss in mammalian cells. Thus, we hypothesized that EC deciliation facilitated by changes in shear stress would manifest in increased abundance of cilia-related proteins in circulation. To test this hypothesis, we performed shear stress experiments that mimicked flow conditions from low to high shear stress in human primary cells and a zebrafish model system. In the primary cells, we showed that upon shear stress induction, indeed, ciliary fragments were observed in the effluent in vitro, and effluents contained ciliary proteins normally expressed in both endothelial and epithelial cells. In zebrafish, upon shear stress induction, fewer cilia-expressing ECs were observed. To test the translational relevance of these findings, we investigated our hypothesis using patient blood samples from sickle cell disease and found that plasma levels of ciliary proteins were elevated compared with healthy controls. Further, sickled red blood cells demonstrated high levels of ciliary protein (ARL13b) on their surface after adhesion to brain ECs. Brain ECs postinteraction with sickle RBCs showed high reactive oxygen species (ROS) levels. Attenuating ROS levels in brain ECs decreased cilia protein levels on RBCs and rescued ciliary protein levels in brain ECs. Collectively, these data suggest that cilia and ciliary proteins in circulation are detectable under various altered-flow conditions, which could serve as a surrogate biomarker of the damaged endothelium.

## Graphical abstract

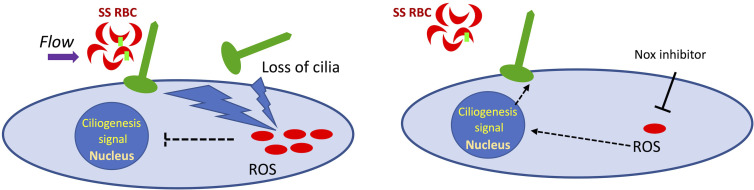

## Introduction

Cilia are microtubule-based organelles that are present in most eukaryotic cells (1) and are distinguished based on the arrangement of 9 outer microtubule doublets enclosing a central doublet (9+2) or not (9+0). 9+0 cilia are often referred to as primary nonmotile cilia and 9+2 cilia as motile cilia (2). However, these definitions need revisiting given the recent evidence suggesting that mixed cilia (motile and nonmotile) can be found in eukaryotic cells (3). Cilia function as mechanosensors for cells. In endothelial cells (ECs) that line the vasculature, the 9+0 cilia are often found on the apical (luminal) surface and are thought to sense blood flow and transduce these mechanical signals into chemical signals inside the cells that control shear-responsive behavior (4, 5). Primary endothelial cilia’s role as mechanosensors has been reported in mouse aortic ECs in vitro, isolated mouse arteries, blood vessels from human placenta ex vivo, and in vivo mouse models (6–10). Shear stress from physiologic blood flow elicits cilia bending (11). Shear stress, the tangential force of blood flow on the surface of the endothelium, normally varies throughout the macro- and microvasculature and can drastically vary in different physiologic and pathologic conditions. Ciliary length is often correlated with mechanosensory action in blood vessels, with cells experiencing low shear stress having longer cilia and cells in blood vessels with high shear stress having shorter cilia or no cilia (12, 13). In both zebrafish and mammals, primary cilia are considered enriched in regions of low shear stress (11, 14). At low shear stress, in the mammalian retinal vasculature, primary cilia are suggested to act in concert with bone morphogenetic protein 9 to minimize vessel regression before onset of high shear stress–mediated vascular remodeling (15). Thus, collectively, EC cilia are widely considered as low shear stress sensors.

In our own work, we have corroborated some of these initial findings and identified EC cilia in regions of low shear stress, such as bifurcation junctions in the juvenile zebrafish vasculature (16). Thus, the question arises as to what happens to EC cilia under increased shear stress conditions. Seminal work from Iomini et al. shows disassembly of primary cilia from human umbilical vein ECs (HUVECs) experiencing laminar shear stress of 15 dyne/cm^2^ for 1 hour (13). Cilia consist of an axoneme that projects into the lumen and are anchored to the cell through transition fibers that connect to the basal body, a centriole-derived structure (1). Recent work suggests that cilia can be physically removed from mammalian cells, a process called deciliation, which releases the ciliary membrane and axoneme from the basal body (17). Intact shed cilia were recovered in culture media and contained both membrane and axoneme fragments. Further, this deciliation process was reported to be rapid and was suggested as the predominant mode of cilia loss in mammalian cells. In a separate study, whole cilia or partial ciliary fragments were observed in urine from mice subjected to chemically induced acute kidney injury (18) presumably from kidney epithelial cells, thus attributing this phenomenon to multiple cell types and tissues. The consequence of high shear stress–induced deciliation in embryonic ECs is directly associated with vascular instability and hemorrhage in embryonic zebrafish (16, 19–21) and vascular barrier integrity (22, 23). In mice (24, 25), ciliary mutants show extensive hemorrhages. These studies collectively suggest the hypothesis that cilia in ECs are disassembled (deciliation) upon disruption of vascular homeostasis, thus causing release of the ciliary fragments into circulation. Detection or quantification of ciliary proteins in circulation may serve as a biomarker for pathologic conditions of altered flow or changes in blood viscosity. In this study, we tested this hypothesis using a combination of cellular, vertebrate, and human model systems.

## Results

### Increased shear stress in ECs facilitates deciliation in vitro.

**Figure 1:**
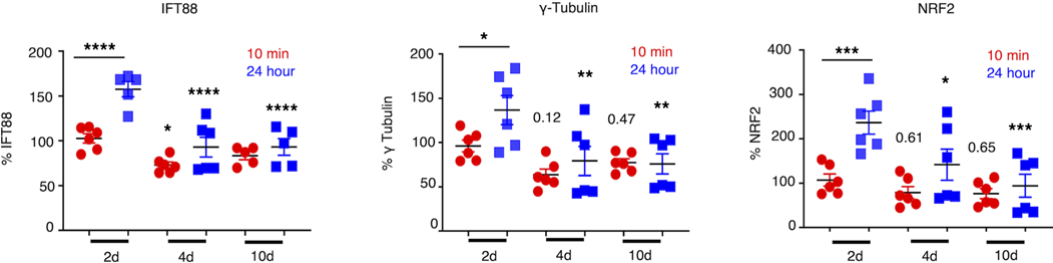
Shear stress causes brain ECs to express fewer cilia proteins in vitro. HBMVECs were subjected to graded strengths of shear stress (2 dyne/cm^2^, 4 dyne/cm^2^, and 10 dyne/cm^2^) by the Ibidi flow system. A total of 2 dyne/cm^2^ was utilized as a “steady-state” flow condition. Following flow with durations as indicated, the expression of cilia-associated proteins was quantified as MFI by flow cytometry. NRF2, the transcription factor reported to control cilia formation and function, was also included in the study. Expression of proteins in the samples was normalized against their respective “no flow” controls. ANOVA was performed to compare between the experimental groups versus steady-state control group for 10-minute or 24-hour time points. ANOVAs were 2 way. Analysis was also performed between 10 minutes and 24 hours in 2 dyne/cm^2^ group. In all 3 protein expressions, no statistical difference was observed between 4 dyne/cm^2^ and 10 dyne/cm^2^ groups. *P < 0.05, **P < 0.01, ***P < 0.001, ****P < 0.0001. For all groups reported in this figure, *n* = 6 except for IFT88 (*n* = 5 for 10 dyne group).

To test the effect of shear stress on EC cilia in vitro, we chose cells from a microvascular brain vessel bed, human primary brain microvascular ECs (HBMVECs), and a macrovessel venous bed, HUVECs. We applied laminar flow shear stress of 0, 2, 4, and 10 dyne/cm^2^ on HBMVECs, which was reported previously (26), and used 2 different methods for subjecting the cells to shear stress. In a microfluidic device–based unidirectional shear stress method (Ibidi system), we used graded strengths of shear stress starting at 2 dyne/cm^2^ and increased to 4 and 10 dyne/cm^2^ for 10 minutes and 24 hours (Figure 1). With a shaker method, which is based on circulatory motion-associated shear stress, we applied 4 and 10 dyne/cm^2^ for 4 minutes (Supplemental Figure 2; supplemental material available online with this article; https://doi.org/10.1172/jci.insight.151813DS1). After shear stress, cells were collected for FACS analysis of specific markers: KLF4, KLF2, ALK1 (flow), ARL13b (cilia axoneme), γ-tubulin (cilia basal body), IFT88 (cilia transition zone protein), Inversin (cilia basal body), NRF2 (cilia-related gene), and bACTIN (housekeeping gene) (Supplemental Figure 3A). With both methods, we observed the control flow marker expression increase in brain ECs. With the Ibidi method, KLF4 (Supplemental Figure 1A) and KLF2 (Supplemental Figure 1B) expression were significantly increased in 10 dyne/cm^2^ condition compared with the “no flow” control at 24 hours. With the shaker method, a linear increase in ALK1-expressing cells at 4 and 10 dyne/cm^2^ (*P* < 0.0001) (Supplemental Figure 2A) was observed.

With both methods, interestingly, all ciliary markers tested showed reduction in protein levels at 10 dyne/cm^2^ and 4 dyne/cm^2^ (Figure 1 and Supplemental Figure 2, C–F), with no change in housekeeping bACTIN protein expression (Supplemental Figure 2B). With Ibidi method, at a shorter period of 10 minutes, compared with 24 hours, lower protein expression levels were generally observed (Figure 1), which was distinct at 2 dyne/cm^2^, a physiologically steady-state situation. At 10 minutes as well as 24 hours, the 4 and 10 dyne/cm^2^ groups showed significantly reduced cilia-associated protein expression compared with the steady-state control group. Compared with steady-state controls, experimental groups (4 and 10 dyne/cm^2^) had about 20% less expression of any cilia-associated proteins after 10-minute perfusion (Figure 1). The reduction of protein expression in the groups was even pronounced (>50%) at 24 hours postperfusion (Figure 1).

**Figure 2:**
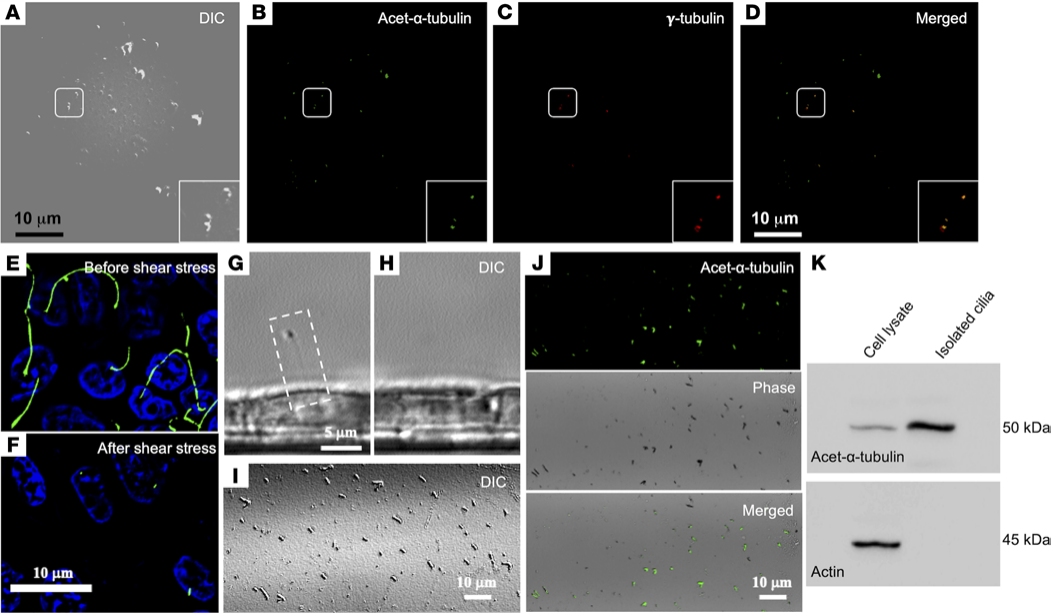
Shear stress causes deciliation of endothelial and epithelial cells in vitro. (**A**–**D**) HUVECs grown on monolayer. Fluid shear stress of 20 dyne/cm^2^ was perfused onto the cells, and the perfusate was collected. The drop concentrated perfusate was analyzed with DIC microscopy and stained with markers for cilia (acetylated-α-tubulin) (**B**) and basal body (γ-tubulin) (**C**). Enlarged images are also shown in the boxes. (**D**) Merged image is shown. (**E** and **F**) Immunostaining (acetylated-α-tubulin, green, cilia; and DAPI, blue, nucleus) for the presence and absence of the cilia from the epithelial cell population before (upper panel) and after (lower panel) the application of 10 dyne/cm^2^ shear stress, respectively. (**G** and **H**) Phase contrast DIC image of a primary cilium in a single live cell (white dotted box). The same cell was imaged before and after 4-minute application of 10 dyne/cm^2^ fluid shear stress. (**I**) Perfusate under DIC microscopy. (**J**) A separate experiment, where perfusate was collected and stained with ciliary marker (acetylated-α-tubulin; green) to confirm the presence of cilia using both fluorescence and phase contrast imaging (**I**). (**K**) Cilia and cell lysates were immunoblotted with cilia marker (acetylated-α-tubulin) to molecularly confirm the presence of the cilia in the perfusate (*n* = 6). **E**–**K** represent porcine kidney epithelial cells (LLC-PK1).

For HUVECs plated on a monolayer, we used the shaker method to apply high shear stress at 20 dyne/cm^2^ as reported previously in the literature (27, 28). Effluents were collected, drop concentrated, and analyzed by differential interference contrast (DIC) microscopy. Staining was performed on the effluents for ciliary marker acetylated-α-tubulin and basal body (γ-tubulin). As shown (Figure 2, A–D), the effluents showed positivity for both acetylated-α-tubulin (Figure 2B) and γ-tubulin (Figure 2C) proteins. We also applied 10 dyne/cm^2^ shear stress to a porcine kidney epithelial cell line, LLC-PK1, using the shaker method and stained for ciliary marker acetylated-α-tubulin (Figure 2, E and F), which clearly showed loss of cilia marker staining after shear stress. A single cilium observed by phase contrast microscopy (Figure 2G, white box) was missing after shear stress (Figure 2H). Further, effluents (Figure 2I) collected from these experiments were positive for acetylated-α-tubulin (Figure 2J), and Western blots for acetylated-α-tubulin clearly showed expression in isolated cilia with no actin protein detected in the same isolate (Figure 2K). Thus, collectively, these data argue that shear stress of greater than 2 dyne/cm^2^ can influence cilia removal, loss of cilia proteins on cell surface, and presence of cilia proteins and fragments in effluents in macro- and microvessel beds as well as kidney epithelial cells.

### Increased shear stress in vivo results in ECs with lower amounts of cilia protein expression.

**Figure 3:**
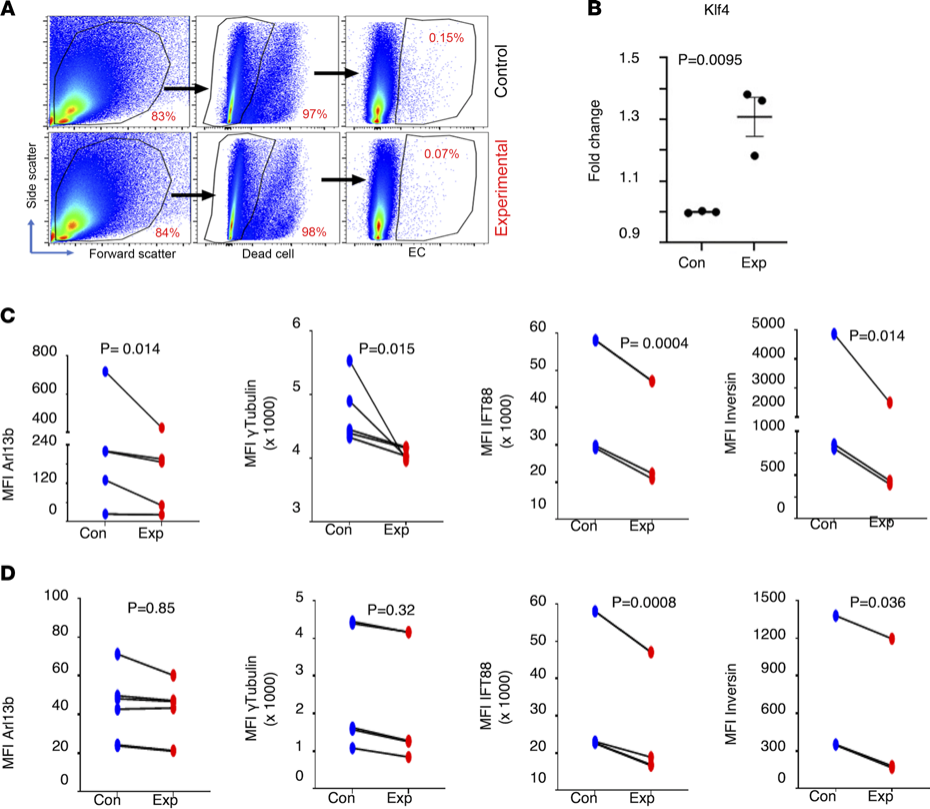
Shear stress results in ECs with fewer cilia proteins in vivo. Flk1^mCherry^ Arl13b^GFP^ double-transgenic zebrafish embryos at 29.5 hpf were subjected to 32°C temperature for 3 hours. Single cells were harvested from dechorionated embryos, and expression of cilia-specific proteins was quantified by flow cytometry in live ECs (mCherry^+^) versus non-ECs (mCherry^–^). Representative dot plots show the gating strategy as applied during FACS analysis to identify ECs (**A**). Stress-responsive protein Klf4 was quantified in ECs (**B**). Protein quantification was done by measuring MFI. Cilia-specific proteins were quantified in ECs (**C**) as well as non-ECs (**D**). Arl13b expression is marked by enhanced green fluorescent protein expression. For Arl13b *n* = 6 (for EC and non-EC); γ-tubulin *n* = 5 (for EC and non-EC); Ift88 *n* = 4 (EC) and *n* = 5 (non-EC); Inversin (*n* = 3 for EC and non-EC); Klf4 *n* = 3 (for EC and non-EC). A linear mixed model was used to examine the differences between treatment group and control group within EC (mCherry^+^) or non-EC (mCherry^–^). Time processed nested within day was treated as random. ARL13b and Inversin expression data were log-transformed to improve fit.

To test the effect of shear stress on EC cilia in vivo, we took advantage of the versatility of the vertebrate zebrafish model system (Supplemental Figure 3B). Previously, others (29, 30) have reported that increasing the incubation temperature of zebrafish embryos from 28°C to 34.5°C increases their heartbeat and blood flow rate. Upon increasing temperature, we calculated that the shear stress increased from 2.3 to 3.1 dyne/cm^2^ in the primordial midbrain venous channel (PMBC) (16) and from 2.0 to 2.97 dyne/cm^2^ in the dorsal aorta (DA). However, these measurements were made at 33 hours postfertilization (hpf), after 5 hours of incubation from 28 to 33 hpf at the higher temperature. We performed a systematic analysis of the effect of temperature (32°C and 35°C) with shorter (3.5 and 5 hours) and longer (24 hours) incubation periods starting at 28 hpf (blood flow commenced in brain), then measured blood flow–associated parameters in the PMBCs and DAs at 48 hpf (Supplemental Figures 4–7). Three groups of embryos (*n* = 20–50 per group) (Supplemental Figure 4) were analyzed in both PMBC (Supplemental Figure 5 and Supplemental Figure 7A) and DA (Supplemental Figure 6 and Supplemental Figure 7B) vessels for blood flow (pulse, blood flow velocity, vessel diameter, shear stress) parameters. The samples included group 1 (G1): 0–48 hpf (28°C); group 2 (G2): 0–28 hpf (28°C) followed by 3.5 or 5 hours of incubation at 32°C or 35°C and returning to 28°C until 48 hpf; and group 3 (G3): 0–28 hpf (28°C) followed by incubation at 32°C or 35°C until 48 hpf (Supplemental Figure 4). We made the following overall observations: (a) the 35°C over 32°C temperature showed more robust changes in blood flow parameters (pulse, velocity, vessel diameter, and shear stress) but also induced scoliosis (curvature of spine) in embryos (Supplemental Figure 8); (b) irrespective of the vessel caliber or location, for the most part, G3 embryos showed higher blood flow parameters; (c) shear stress values were generally higher for PMBC (3–5 dyne/cm^2^ [32°C] or 4–7 dyne/cm^2^ [35°C]) versus DA vessels (2.5–3 dyne/cm^2^ [32°C] or 3.5–5.2 dyne/cm^2^ [35°C]); and (d) exposure for 3.5 hours at 32°C also showed robust changes in the assessed parameters of the PMBC except for blood flow velocity (Supplemental Figure 7A), with no significant changes observed in the DA (Supplemental Figure 7B). It is noteworthy that increasing shear stress by this method only showed hemorrhages in brain vessels and not the trunk, which is thought to be associated with loss of cilia in ECs (16). Thus, we chose the shorter time point of about 3 hours’ incubation (29.5–32.5 hpf) and lower temperature (32°C) for increasing shear stress in PMBCs of transgenic (Tg) (*flk*: mCherry; *bactin*: Arl13b-GFP) zebrafish embryos, where ECs were labeled red and cilia were labeled green. Single-cell suspension and subsequent FACS analysis on live and labeled ECs (Supplemental Figure 3B) were performed as described in the Methods section. Markers assessed included Klf4 (flow), Arl13b (cilia axoneme), γ-tubulin (cilia basal body), Inversin (cilia basal body), and Ift88 (cilia transition zone). In the shear stress induction group (experimental), we observed the expected increase in Klf4-expressing MFI in ECs (Figure 3, A and B). All cilia markers assessed showed a decrease in experimental samples compared with control samples (Figure 3C) when assessed in the mCherry^+^ (EC) population. In the mCherry^–^ (non-EC) population (Figure 3D), the reduction of the respective cilia-associated protein expressions was less pronounced compared with what was observed in the EC population. These results suggest that upon increased shear stress in brain vessels in vertebrate embryos, ECs express fewer ciliary proteins on their cell surface.

### Circulating RBCs adhere to brain ECs and accumulate cilia proteins postadhesion.

**Figure 4:**
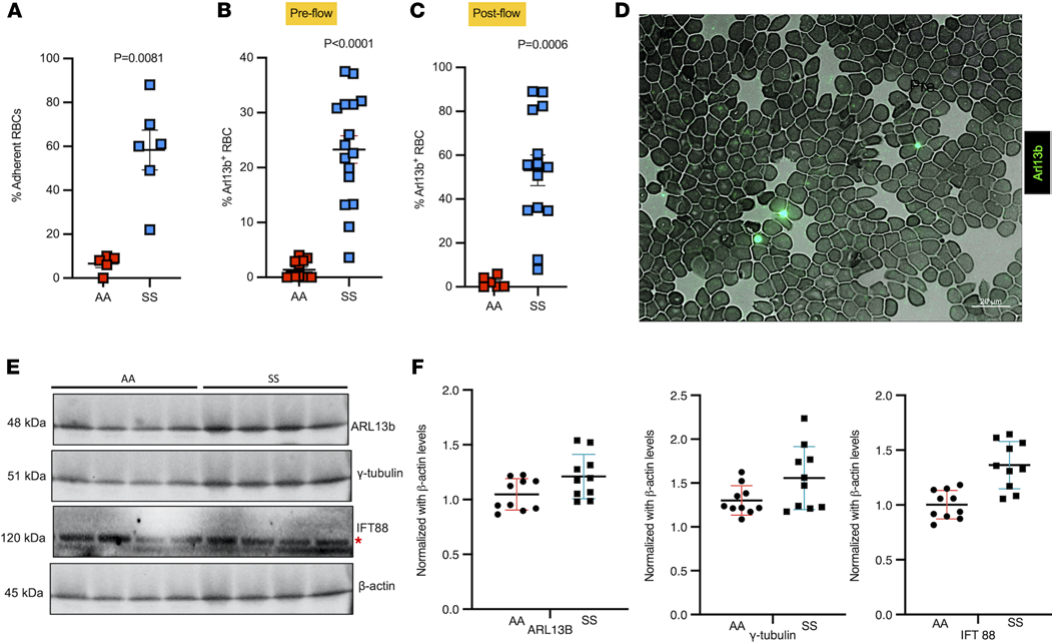
Sickle RBCs adhere to brain ECs triggering deciliation, and cilia are found on sickle RBCs and plasma from SCD. HBMVECs exposed to sickle (SS) or healthy (AA) RBCs were subjected to shear stress (1 dyne/cm^2^), and fraction of SS (*n* = 6) and AA (*n* = 5) RBCs adhered to ECs after stress induction was calculated, *P* = 0.0081 (**A**). SS and AA RBCs were tested for ARL13b cilia prior to flow and proportion of ARL13b cilia adhered to circulating SS RBCs (*n* = 16) versus AA RBCs (*n* = 12) were quantified, *P* < 0.0001 (**B**). After flow, ARL13b expression on SS RBCs (*n* = 11), but not AA RBCs (*n* = 6), upon interaction with ECs, *P* = 0.0006 (**C**). (**A**–**C**) Mann-Whitney-Wilcoxon test *P* values are provided. Representative field (magnification 63×; scale bar = 20 μm) of a smear of SS RBCs shows cilia presence on these sickle cells, detected with FITC-conjugated anti-Arl13b antibody (**D**). Western blot plot shows the detection of cilia-specific proteins in plasma samples of healthy controls (AA) versus sickle (SS). Red asterisk represents the top IFT88 band that was used for quantification (**E**). Please note that Western blots from only 4 AA and SS samples are shown in **E**. A separate gel for the other 6 samples was run and quantified. Quantification includes all 10 samples from each group. Cilia-specific proteins were quantified from plasma samples of healthy controls (AA) (*n* = 10) versus sickle (SS) (*n* = 10) and normalized against housekeeping protein bACTIN (**F**). *P < 0.05, ***P < 0.001. A 2-tailed *t* test or Mann-Whitney-Wilcoxon test was performed to compare between groups.

To investigate ciliary proteins in circulation in a pathophysiologic model with translational and clinical significance, we selected sickle cell disease (SCD). SCD is caused by a single mutation in the *β-globin* gene that changes the sixth amino acid in the β-globin protein of hemoglobin from glutamic acid to valine (31), which makes RBCs highly susceptible to sickling due to the production of sickle hemoglobin (Hb S) and thus impairs Hb capacity to deliver oxygen to tissues. In the cerebral and systemic vasculature, occlusive events in SCD have been postulated to be initiated by sickle RBCs’ adhesion to the endothelium (32). Thus, we hypothesized that sickle RBCs adhere to brain ECs, triggering cilia shedding, and that ciliary proteins would be high in plasma from patients with SCD. We first investigated whether RBCs isolated from patients homozygous for Hb S (SS) would adhere preferentially to a monolayer of brain ECs, compared with healthy volunteers with normal Hb A (AA). A graduated height flow chamber adhesion assay to quantify the adhesion of RBCs to ECs was performed as described previously (33, 34). Indeed, 58% ± 9% of SS RBCs compared with 7% ± 1.8% of AA RBCs adhered to brain ECs subjected to shear stress of 1 dyne/cm^2^ (Figure 4A). Next, we performed flow cytometric analysis on circulating RBCs isolated from SS patients for the presence of cilia protein ARL13b, prior to and following exposure to brain ECs in the flow chamber. Compared with AA RBCs, which showed 1% ± 0.47% of bound ARL13b, SS RBCs showed a remarkable 23-fold increase to 23% ± 2.5% of bound ARL13b prior to exposing brain ECs to the AA and SS RBCs, respectively (Figure 4B). Upon flow exposure, ARL13b presence on AA RBCs was not affected, but it did increase further by 2.3-fold on SS RBCs to 54% ± 8.8% (Figure 4C), suggesting that SS RBCs may have collected additional cilia from brain ECs. We performed a blood smear to directly visualize accumulation of ARL13b on SS RBCs (Figure 4D). A similar blood smear was performed from blood isolated from sickle SS mice and immunostained for ARL13b and IFT88 proteins (Supplemental Figure 9). ARL13b- and IFT88-positive cilia-expressing mouse RBCs were found in smears from SS mice (Supplemental Figure 9, A and B). Quantification (Supplemental Figure 9C) showed a 2-fold enrichment of ARL13b-positive RBCs in SS versus AA mouse blood. Finally, we investigated plasma from 10 patients with SCD and 10 healthy individuals using Western blot for the presence of ciliary proteins ARL13b, γ-tubulin, and IFT88 (Figure 4, E and F). All 3 ciliary proteins were enriched in the plasma from patients with SCD compared with healthy volunteers (Figure 4F). Taken together, these data sets suggest that in a pathologic condition predisposed to vascular occlusion and/or compromised blood flow, ciliary proteins are present on the surface of RBCs, further accumulate on RBCs upon EC contact, and are enriched in plasma. Thus, collectively, our work suggests cilia proteins as potential biomarkers for diagnosis of flow-mediated alterations of the vascular endothelium.

### EC cilia stability is dependent on ROS generation in brain ECs.

**Figure 5:**
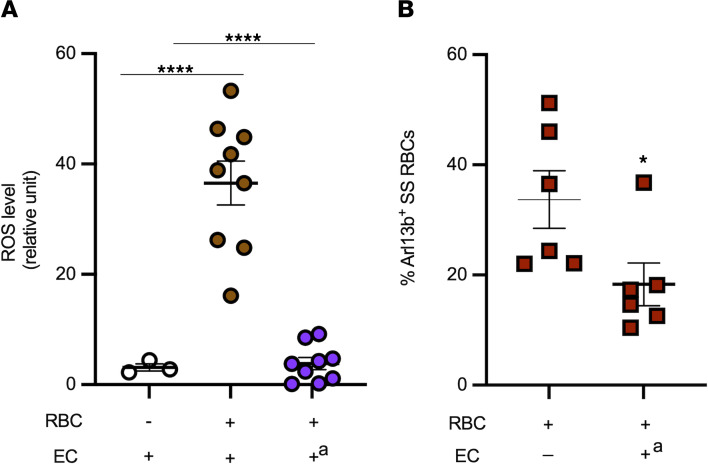
Cilia shedding triggered by sickle RBCs is dependent on RBC-induced increased EC ROS generation. Human brain microvascular ECs were sham treated or treated with the NOX inhibitor apocynin (**A**), prior to exposure or not with sickle RBCs (SS RBCs) (*n* = 6). Exposure of HBMVECs to SS RBCs increased ROS generation in ECs, which is dependent on NOX. Flow cytometry analysis shows ARL13b bound to SS RBCs (*n* = 6) prior to (baseline) and after interaction with shear-stressed ECs pretreated with apocynin (**B**). *P < 0.05, and ****P < 0.0001. +^a^ = ECs treated with apocynin. For **A**, 1-way ANOVA test was performed, and for **B**, Wilcoxon’s signed-rank test was performed.

To investigate the underlying mechanism associated with loss of cilia in brain ECs due to shear stress or RBC interaction, we focused on excessive reactive oxygen species (ROS) and oxidative stress. Previous work has suggested that interactions of sickle RBCs with HUVECs induce endothelial oxidative stress (35). Thus, we investigated whether adhesion of SS RBCs from patients with SCD patients to brain ECs triggers ROS generation in ECs. Indeed, SS RBC interactions with brain ECs increased EC ROS levels compared with basal levels of EC ROS (*P* < 0.05; Figure 5A). Pretreatment of brain ECs with the NADPH oxidase (NOX) inhibitor, apocynin, reduced ROS generation in ECs to baseline levels (*P* < 0.0001; Figure 5A), suggesting that SS RBC–induced increased ROS production in brain ECs is dependent on activation of NOX enzymes. To investigate the effect of increased ROS levels in ECs on SS RBCs-cilia, in subsequent experiments, we analyzed by flow cytometry SS RBCs for the presence of the ciliary protein ARL13b prior to and following exposure in the flow chamber to brain ECs pretreated with apocynin. Before exposure to apocynin-treated brain ECs, SS RBCs showed approximately 35% ARL13b cilia expression, but after exposure to apocynin-treated brain ECs, SS RBCs showed a decrease of approximately 20% in ARL13b expression (*P* < 0.05, Figure 5B). We interpret these data to mean that inhibition of NOX enzymes in brain ECs with apocynin prevented SS RBCs from collecting additional ARL13b protein from brain ECs.

**Figure 6:**
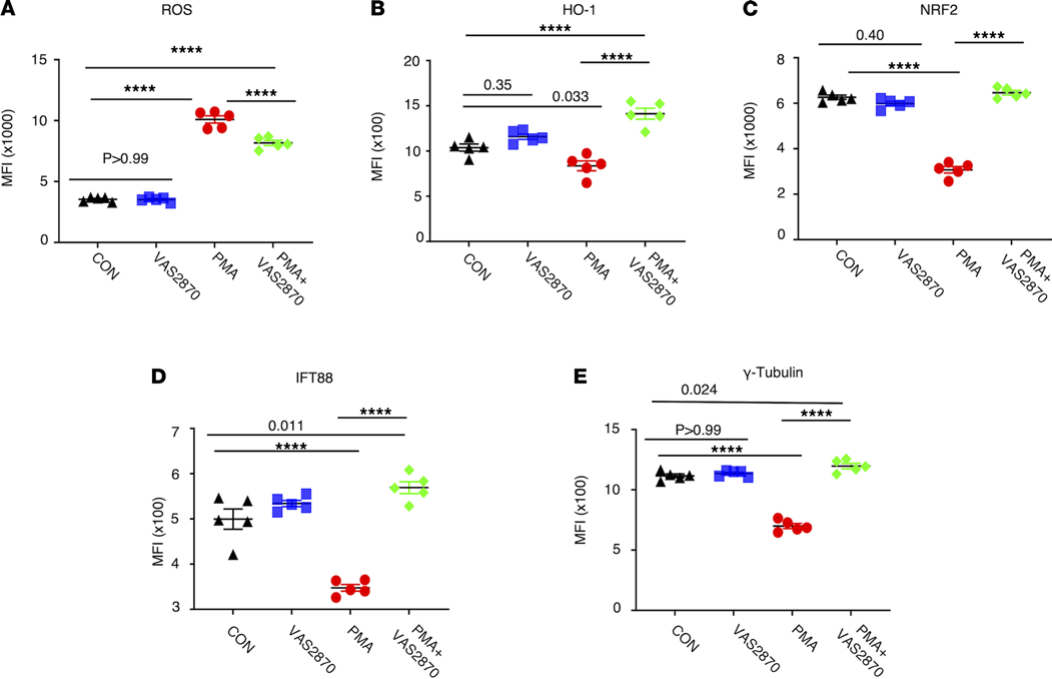
Attenuating ROS production rescues cilia proteins in ECs. HBMVECs were treated with ROS-inducing PMA in presence or absence of NOX inhibitor VAS2870. Untreated groups were also included as controls. ROS production was quantified as MFI by flow cytometry (**A**). Oxidative stress counteracting protein heme oxygenase 1 (HO-1) was quantified (**B**). NRF2, the transcription factor reported to control cilia formation and function, was also included in the study (**C**). Cilia proteins are downregulated by PMA and rescued by NOX inhibitor VAS2870 (**D** and **E**). ****P < 0.0001. ANOVA (1 way) was performed, and Bonferroni’s correction was used to adjust for multiple comparisons (*n* = 5 for all groups).

To assess if attenuating oxidative stress in brain ECs will rescue cilia protein levels, we treated human brain ECs with PMA, a known oxidative stress inducer, in ECs in the presence or absence of the NOX small-molecule inhibitor VAS2870. We then quantified the expression of total ROS, heme oxygenase 1 (HO-1, oxidative stress counteractor), and cilia-associated proteins NRF2, IFT88, and γ-tubulin (Figure 6) levels using flow cytometry method. As expected, PMA induced ROS in brain ECs (Figure 6A), which was partially attenuated by VAS2870. HO-1 levels (Figure 6B) were lower in PMA-treated brain ECs, and upon NOX inhibition, levels were restored to control levels. Interestingly, all 3 cilia-associated proteins (Figure 6, C–E) were reduced upon PMA treatment, and levels were restored to baseline upon NOX inhibition in brain ECs. Taking the sickle RBC ROS data and brain EC–based ROS data together, oxidative stress appears to be one of the key pathways in ECs that is responsible for cilia stability.

## Discussion

Our present study reveals that cilia-specific proteins can be quantified in the plasma and should be explored as biomarkers of endothelial damage or dysfunction. In the ECs lining the vascular wall, cilia have been postulated as a low-flow sensor (11). In support of this hypothesis, cilia are indeed enriched in regions of vessel wall where flow is minimal, such as curvature in the vessel (14, 16). Under steady state, the vasculature experiences a constant flow. In the venous system, an average shear force of 1–4 dyne/cm^2^ is reported while capillaries experience shear from 10–20 dyne/cm^2^ (26). In arteries, shear force varies from 4 dyne/cm^2^ in the common carotid artery to 13 dyne/cm^2^ in the brachial artery (36). We previously demonstrated in zebrafish embryos, that upon incubation of embryos at 34.5°C, 2–4 dyne/cm^2^ shear stress is induced in brain microvessels that results in brain hemorrhage and loss of ciliary structures in primordial midbrain venous channels (16). Others have also reported that loss of ciliary proteins in zebrafish causes brain vessel instability resulting in hemorrhages (19). Further, primary cilia are disassembled when HUVECs (macrovessels) are subjected to 15 dyne/cm^2^ of laminar shear stress (13). Thus, collectively, 4–20 dyne/cm^2^ shear force as observed in vivo is sufficient to make ECs lose ciliary proteins in vitro and was indeed observed in this study. Here, we were able to detect ciliary fragments in effluents in cell culture from ECs and epithelial cells and were able to detect fewer cilia-expressing cells in vivo in ECs subjected to high shear stress.

**Figure 7:**
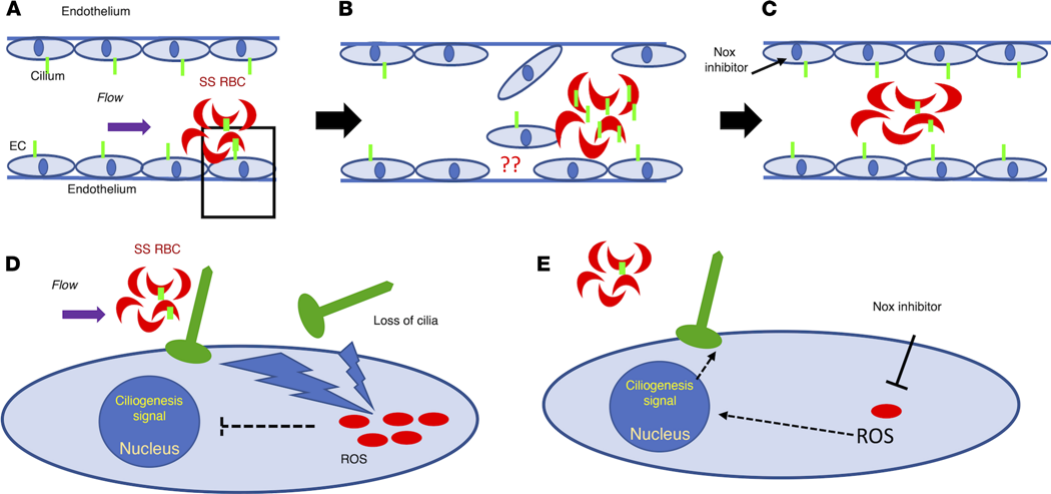
Proposed mechanism of loss of cilia proteins. The schematic demonstrates cilia stability mechanisms associated with sickle RBCs (SS RBCs) and brain ECs in an SCD setting. Green-colored structures on RBCs and ECs are cilia, and red-colored dots in (**D** and **E**) are oxidative stress components, such as ROS. SS RBCs create altered shear stress on ECs due to their sickle shape. Upon interaction with ECs, SS RBCs elevate endothelial oxidative stress (**A** and **D**). The physical (shear) or metabolic (oxidative) stress results in loss of endothelial cilia or cilia-associated proteins. These shredded cilia fragments (and associated proteins) are now available for interaction with SS RBCs and may enrich the RBCs’ surface (**B**). Upon attenuation of oxidative stress in ECs (NOX inhibitor), the loss of endothelial cilia or any subsequent enrichment of cilia in sickle RBCs is minimized (**C**, fewer green cilia in RBC compared with in **B**). (**D** and **E**) Magnified images of **A**, which details the mechanism identified in this study. Sickle RBCs upon adhering to ECs elevate oxidative stress (increased ROS) in ECs (**D**). This causes loss of cilia or cilia-associated protein and may send inhibitory signals to the nucleus to prevent a ciliogenesis signal. Once the oxidative stress is attenuated by NOX inhibition, ROS is downregulated, and restoration of endothelial cilia is observed (**E**).

The question of how cilia on the EC surface may be influenced by altered flow to facilitate detachment remained an open question. In addition to increased or disturbed physical flow, we hypothesized that blood constituents, in particular, RBCs, may also contribute to the deciliation process in ECs (17) in pathologic conditions (Figure 7). We chose SCD, where the RBC morphology is altered to have a sickled shape and RBCs are known to cause oxidative stress–mediated damage to EC membranes (35, 37, 38). Further, in SCD, blood flow is altered because of enhanced adhesion of sickle RBCs to themselves and the endothelium, promoting vascular occlusion (39), which can subsequently facilitate damage to the endothelium (35). Thus, this pathophysiologic model offers the ideal opportunity to identify cilia proteins emerging from the damaged endothelium that may be the result of sickle RBC adhesion to the endothelium. Remarkably, we found that human sickle RBCs displayed enhanced ARL13b ciliary protein on their surface compared with normal RBCs, and once they encountered brain ECs under intermittent flow conditions, the presence of the ciliary protein on these sickle cells was 2.3-fold higher. These results concur with the detection of ciliary proteins at higher levels in SCD plasma compared with healthy plasma. Interestingly, we observed all 3 representative components of the ciliary structure — namely axoneme, transition zone, and basal body — in the plasma of patients with SCD, suggesting that cilia disassembly is not partial. These results also concur with the recent observations in mammalian cells, where cilia shed from cells expressing mCherry-atubulin (axoneme marker) contained atubulin in the ciliary fragments, suggesting that axoneme is shed together with the ciliary membrane (17).

To investigate the underlying mechanism associated with cilia stability in static and flow-induced sickle RBCs/brain ECs interaction, we focused on oxidative stress and ROS generation (Figure 7). Human sickle RBCs adhere to brain ECs similar to HUVECs (35) and induce increased ROS generation. Sickle RBCs exposed to brain ECs treated with apocynin, a NOX inhibitor, showed decreased cilia protein expression. These data argue that cilia on sickle RBCs are influenced upon interaction with ROS-quenched brain ECs (Figure 7). Two interrelated possibilities emerge to explain this result: either ECs retain more cilia when oxidative stress is minimized and thereby RBCs capture fewer cilia from ECs, or RBCs lose cilia when they interact with ROS-inhibited ECs and thus have fewer cilia. It is difficult to differentiate between the two. Under static conditions, brain ECs were responsive to stress inducers such as PMA, and showed enhanced total ROS, which upon treatment with a different NOX inhibitor, VAS2870, quenched ROS production following the PMA-induced oxidative stress. Interestingly, the cilia protein levels that were decreased in PMA-treated brain ECs returned to baseline in NOX inhibitor–treated brain ECs. The static condition result argues against the possibility that ECs’ cilia are not available for interaction upon ROS inhibition and supports the second hypothesis that RBCs lose cilia upon interaction with ROS-inhibited ECs. These results collectively argue that cilia stability in both ECs and RBCs is susceptible to increased ROS levels in brain ECs. This interpretation is also in line with previous studies in epithelial cells where they show that, after ischemic injury in murine kidneys, reduction of oxidative stress accelerates the recovery of primary cilia length (40).

For SCD, the identification of enhanced cilia protein on RBCs may prognosticate adverse events such as vascular wall weakening, and susceptibility for hemorrhage, both clinically relevant features observed in patients with SCD. Having this information is beneficial for making informed clinical decisions. Because ECs experience altered flow or shear stress in various pathophysiologic conditions, such as preeclampsia, polycystic kidney disease, hypertension, and stroke to name a few, circulating ECs have been considered as possible biomarkers of vascular insult or endothelial dysfunction (41, 42). However, due to their low numbers in circulation, and difficulty in detection, their application has been limited. On the other hand, as shown here, cilia from damaged ECs can be detected readily in circulation. This method is perhaps a better alternative to detecting circulating ECs given that blood components are the only point of contact for EC cilia expressed on the luminal side. Also, given that cilia are expressed in most EC beds and that flow influences cilia integrity, any condition where flow is compromised constitutes an opportunity for the application of cilia biomarkers, broadening the value of cilia biomarkers for vascular injury. Some key questions remain that will dictate the development of this concept. For example, can the cilia protein increase in peripheral blood inform us of the source of the injured vessel bed (i.e., cerebral vascular bed, vs. pulmonary capillary vs. coronary capillary)? Are there unique cilia protein signatures or cilia-associated cells in peripheral blood that itself could serve as trackers of a condition or of response to a particular treatment? Answers to these questions will guide the effectiveness of cilia as a biomarker that can predict and evaluate the state of a disease. Nevertheless, at minimum, the ability to detect ciliary proteins in blood or other body fluids and in various conditions influenced by flow will provide an additional tool in the clinical toolbox to inform the physician of a possible pathology. In summary, our work warrants extended investigation to understand whether and how cilia-specific proteins in circulation can be developed into prognosticative markers of disease where the flow-related homeostasis of the endothelium is compromised.

## Methods

### Antibodies

Primary antibodies used in this study include ARL13b (Proteintech catalog 17711-I-AP), acetylated tubulin (Sigma catalog T6793), IFT88 (Thermo Fisher Scientific catalog PA5-18467), Inversin (Proteintech catalog 10585-I-AP), Dynein (Thermo Fisher Scientific catalog MA1-070), gTubulin (GeneTex catalog GTX113286), Alk1 (Abcam catalog ab51870), KLF4 (Proteintech catalog 11880-I-AP), HO-1 (BD, catalog 566391), NRF2 (BioLegend, catalog 939202), and bactin (Sigma catalog A5441 and Cell Signaling Technology catalog 4970P). Secondary antibodies used are goat anti-mouse PECy7 (BioLegend), donkey anti-rabbit PE (Thermo Fisher Scientific), donkey anti-goat AF657 (Thermo Fisher Scientific), donkey anti-rabbit BV421 (BioLegend), and donkey anti-rabbit AF488 (Thermo Fisher Scientific).

### Cell culture

Primary HBMVECs (Cell Systems Corporation catalog ACBRI 376) and HUVECs (Glyco Tech) were maintained at 37°C in a 5% CO_2_ incubator in endothelial cell complete medium (Promocell, catalog C22010). As per vendor’s description, cells were isolated from the cortex region of the brain from a pediatric male donor. The cells were isolated without using antibody labeling to preserve the cells’ natural properties for enhanced biological relevance. LL-CPK1 (CL101.1TM) porcine renal epithelial cells from proximal tubule were obtained from ATCC. All cell culture wells were seeded equally, and wells were randomized to control versus experimental conditions with duplicates or triplicates per condition. All experiments were performed between passages 4 and 6. Cells that had reached approximately 90% confluence were used in the shear stress experiment. For ROS quantification experiments, cells were treated with PMA (Sigma, catalog P8139-5MG) at a concentration of 50 ng/mL for 1 hour. To inhibit ROS production in respective experimental groups, cells were treated with 20 μM NOX inhibitor VAS 2870 (Sigma, catalog SML0273-5MG) 1 hour prior to PMA treatment.

### Human patient studies

Blood samples were collected from adult patients with SCD homozygous for Hb S and from healthy adult donors. Patients and donors were recruited under the study protocol approved at Duke University. All patients with SCD had not been transfused for at least 3 months and had not experienced an acute vaso-occlusive crisis for the past 3 weeks, and 98% of the patients tested were on hydroxyurea. Blood samples were collected into citrate tubes. All RBCs were washed in PBS with Ca^2+^ and Mg^2+^ with collection of the plasma and removal of buffy coat.

### Western blot

Plasma isolated from blood collected from patients with SCD (SS) and healthy (AA) volunteers was used for quantification of the following proteins: ARL13b, IFT88, and γ-tubulin. These samples were run on SDS-PAGE, and traditional Western blot protocols were performed. Primary antibodies used were explained before. Secondary antibodies used include anti-rabbit HRP (catalog 7074, Cell Signaling Technology) and anti-mouse HRP (catalog 7076, Cell Signaling Technology). Quantification was done using ImageJ software (NIH) and plotted against the housekeeping control protein bACTIN.

### In vitro EC shear stress experiments

#### Ibidi perfusion.

To generate shear stress in vitro, we utilized the Ibidi pump system. Prior to initiating perfusion, HBMVECs were seeded onto a μ-slide (0.6 mm channel height; Ibidi, 81106) at 0.5 × 10^6^ cells per slide. Slides, media, and a perfusion set (red, 10962) were incubated in a humidified cell culture incubation chamber (37°C in 5% CO_2_) overnight prior to perfusion to prevent bubble formation, per manufacturer recommendations. Immediately prior to perfusion, medium was added to the syringe reservoirs (12 mL total) and air bubbles were removed. The μ-slide was attached to the perfusion set under sterile conditions, and then the fluidic unit was connected to the Ibidi pump and air tubing inside the cell culture incubation chamber. Using the vendor-specific PumpControl software, HBMVECs were perfused at 0, 2, 4, or 10 dyne/cm^2^ for 10 minutes or 24 hours. A total of 2 dyne/cm^2^ was included as representative of steady state that mimics in vivo condition. To prevent acute cell detachment upon initiation of high shear stress at 10 dyne/cm^2^, cells were acclimatized to flow at 4 dyne/cm^2^ for 5 minutes and then subjected to flow at 10 dyne/cm^2^ for either 10 minutes or 24 hours. For experiments at 2 and 4 dyne/cm^2^, no acclimatization was necessary, and cells were subjected to flow for 10 minutes or 24 hours. Per manufacturer recommendations with the 0.6 mm μ-slides and red perfusion sets, the minimum shear stress possible was 2.3 dyne/cm^2^ (rounded off to and referred to as 2 dyne/cm^2^). Immediately following perfusion, cells were trypsinized with TrypLE express (Thermo Fisher Scientific, 12604013) and used for downstream analysis. All the samples subjected to any magnitude of perfusion had their respective no flow controls. Any protein expression in a given sample was normalized against that of no flow control.

### Shaker method

A shaking incubator (New Brunswick Scientific) was used as previously described to induce shear stress (43). The formula used applied to calculate the stress is: shear stress = (6 × F × m)/(w × h^2^), where F = moment of inertia (i.e., function of centrifugal force based on rpm and size of the shaker), m = viscosity of the fluid (i.e., function of temperature), w = diameter of plates (i.e., function of area of dish), and h = height of the fluid (i.e., function of volume). 

A 100 mm culture dish (confluent with cells) that was placed on an orbital shaker at 240 rotations per minute (RPM) resulted in a shear stress of 10 dyne/cm^2^ and at 96 RPM resulted in a shear stress of 4 dyne/cm^2^. The cells were under shear stress for 4 minutes.

For HUVEC and epithelial cell shear stress experiments, the deciliation was induced by mechanical force, as previously described (43, 44). Cell populations were first rinsed briefly and gently with 10 mL of PBS (pH 7.4). A 150 mm culture dish was placed in a flow chamber as previously discussed (6). A shear stress of 10 or 20 dyne/cm^2^ was applied to the cell for 4 minutes. The media containing the excised cilia were carefully transferred to a 50 mL centrifuge tube and centrifuged for 30 minutes at 3000*g* at 4°C. The supernatant containing the excised cilia was then transferred to a polyallomer tube and centrifuged for 1 hour at 70,000*g* at 4°C in an ultracentrifuge. The purified primary cilia were then resuspended in the PBS buffer or RIPA buffer for further analyses.

### Quantitative reverse-transcription PCR

Total RNA was extracted using TRI Reagent and Direct-zol RNA Miniprep (Zymo Research, R2051). RNA was reverse-transcribed into cDNA using 250 ng total RNA (iScript gDNA clear cDNA synthesis kit; BioRad, 172-5034). RNA levels were quantified using custom-designed primers (Primer3) for *KLF2*, *KLF4*, and *GAPDH*. cDNA and primers were mixed with iTaq Universal SYBR Green Supermix (BioRad, 172-5121) and run with the following cycling protocol: 95°C for 2:00 minutes, followed by 40 cycles of 95°C for 0:10 minutes and 60°C for 0:30 minutes (BioRad, CFX96 Real-Time System). Quantification of gene expression was performed using the 2-ΔΔCT method (45). Samples were all run in quadruplicate, and target genes were normalized to GAPDH.

Primers were *KLF2* (133 bp)—forward: CACCAAGAGTTCGCATCTGA, reverse: CGTGTGCTTTCGGTAGTGG; *KLF4* (132 bp)—forward: CGGCTGTGGATGGAAATTCG, reverse: ATGTGTAAGGCGAGGTGGTC; and *GAPDH* (128 bp)—forward: CCAAGGAGTAAGACCCCTGG, reverse: CAACTGTGAGGAGGGGAGAT.

### In vitro adhesion assays

HBMVECs were cultured until they reached confluence on clear glass slides precoated with 2% gelatin. Slides coated with brain ECs were washed, then fit into a variable height flow chamber and tested for their ability to support adhesion of RBCs. The flow chamber was mounted on the stage of an inverted phase contrast microscope (Diaphot, Nikon Inc.) connected to a thermoplate (Tokai Hit Co., Ltd.) set at 37°C. Fluorescence-labeled RBCs suspended at 0.2% (v/v) in PBS with Ca^2+^ and Mg^2+^ were infused into the flow chamber and allowed to adhere to brain ECs for 10 minutes without flow. Before exposure to flow, a minimum of 3 fields at each of 7 different locations along a line oriented to future flow were examined, and the total number of fluorescent cells was counted. Fluid flow (PBS with Ca^2+^ and Mg^2+^) with a calibrated syringe pump was then started for a period of 15 minutes. Effluent was collected and tested by flow cytometry for cilia shedding from brain ECs by RBC contact. After exposure to flow, the fields were examined, and the number of fluorescent adherent RBCs to brain ECs was counted. The fraction of adherent cells was presented as number of cells attached per field after exposure to flow/total number of cells present per field before flow.

The wall shear stress was calculated as t_w_ = 6Qm/wh^2^, where t_w_ = wall shear stress (dyne/cm^2^); Q = volumetric flow rate (cm^3^/s); m = media viscosity, w = width of the flow channel, and h = height of the flow chamber as a function of position along the microscope slide. Blood flow in small vessels may be continuous (nonpulsatile) with shear stresses of 1–2 dyne/cm^2^, or flow may be intermittent (pulsatile). Our data were obtained using pulsatile flow conditions.

### Shear stress zebrafish flow parameter measurements

The transgenic line *Tg(bact:Arl13b-GFP)* was obtained from Brian Ciruna (University of Toronto, Toronto, Ontario, Canada). *Tg(kdrl:mCherry-CAAX)*, Casper, and wild-type AB lines were obtained from Zebrafish Informational Resource Center. Embryos from the wild-type (AB) strain were used in this study. Freshly fertilized embryos were procured through natural breeding of adult zebrafish and were raised at 28.0°C in E3 medium containing 0.1 mM *N*-Phenylthiourea (PTU; Sigma) to inhibit pigment formation. For shear stress experiments, at 28 hpf, fish embryos were divided into 3 groups: G1: 0–48 hpf (28°C); G2: 0–28 hpf (28°C) followed by 3.5 or 5 hours of incubation at 32°C or 35°C and returning to 28°C until 48 hpf; and G3: 0–28 hpf (28°C) followed by incubation at 32°C or 35°C until 48 hpf (Supplemental Figure 4). Control and experimental embryos were subsequently dechorionated at 48 hpf for imaging. A stereomicroscope (Zeiss SteREO Discovery V12 Microscope equipped with Hamamatsu Orca Flash high-speed camera and a workstation equipped with HCImage software, Hamamatsu Photonics) was used to visualize the blood vessels of zebrafish embryos as previously described (46). High-speed video microscopy movies of the heart and tail in 1000 frames per 10 seconds at 100× magnification were recorded for the PMBCs and the DA. The recorded movies were analyzed according to our previous protocols (47, 48) using MicroZebraLab blood flow from Viewpoint (version 3.4.4), and 4 cardiac parameters were calculated for each vessel: pulse, blood flow velocity, vessel diameter, and shear stress using the following formula: τ = 4μV_mean_/D, where μ = blood viscosity (dyne/cm^2^), V = average blood velocity (μm/s), and D = vessel diameter (μm).

This experiment was performed multiple times, and data from 3 independent experiments are reported.

### Whole-mount staining of Casper zebrafish using O-dianisidine

Embryos from Casper strain were used to visualize hemorrhage in the brain after the induction of shear stress as described in the section above. At 48 hpf, embryos were dechorionated and stained with O-dianisidine (stains RBCs). The stain was prepared by mixing 0.6 mg/mL of O-dianisidine (Sigma), 0.65% hydrogen peroxide, 0.01 M sodium acetate (pH 4.5), and 40% (v/v) ethanol solution. Embryos were washed with PTU-E3 medium, which is *N*-Phenylthiourea dissolved in egg water made in-house, and then 0.6 mg/mL of the stain was added and left for 15 minutes in the dark. After staining, embryos were postfixed in 4% paraformaldehyde at 4°C for at least 1 hour. Three percent (w/v) methyl cellulose was used to fix the embryos on the slide for imaging under a bright-field microscope (Stemi 508, Zeiss). Zeiss AxioCam ERc 5s professional digital camera was used for imaging.

### In vivo (zebrafish) shear stress experiments

Embryos from a cross of *Tg(bact:Arl13b-GFP)* and *Tg(kdrl:mCherry-CAAX)* were used in this study (Supplemental Figure 3B). Freshly fertilized embryos were procured through natural breeding of adult zebrafish and were kept at 28.0°C in 1× E3 embryo medium (E3 medium) containing 5 mmol/L NaCl, 0.17 mmol/L KCl, 0.33 mmol/L CaCl_2_, 0.33 mmol/L MgSO_4_, and 0.05% methylene blue. For shear stress experiments, at 29.5 hpf, fish embryos were transferred to an incubator at 32.0°C for 3 hours. Control and experimental embryos were subsequently dechorionated and digested to yield single cells (Supplemental Figure 3B). The composition of digestion buffer used was RPMI 1640 medium (Thermo Fisher Scientific) supplemented with 10% FCS, collagenase D (1 mg/mL), and DNase I (10 μg/mL). Embryos were digested for 30 minutes at 37°C and subsequently passed through a 70 μm cell strainer. Cells were centrifuged at 300*g* for 5 minutes and washed twice with PBS before use for downstream applications. This experiment was performed multiple times, and data from 3 independent experiments are reported.

### Flow cytometry

Single-cell suspensions were washed 3 times with FACS buffer (1× PBS with 5% FBS and 0.1% NaN_3_) at 300*g* for 5 minutes and were subsequently incubated with Live/Dead fixable yellow dead cell stain as per manufacturer’s protocol, to exclude any dead cells, wherever applicable. Then, cells were fixed and permeabilized using Cytofix/Cytoperm buffer (BD, catalog 554722) or transcription factor buffer set (BD, catalog 562574) and stained for the following proteins: ARL13b, IFT88, Inversin, Dynein, γ-tubulin, Alk1, KLF4, HO-1, NRF2, and bACTIN. Suitable secondary reagents were used to detect the respective proteins. Primary antibodies were diluted 1:50, and secondary antibodies were diluted 1:500. BD perm wash buffer (catalog 554723) was used for antibody dilutions and washing. Primary antibodies were incubated for 45 minutes and secondaries for 30 minutes at 4°C. Suitable secondary antibody controls were included. To quantify total ROS, assay kit was used as per the manufacturer’s instruction (Thermo Fisher Scientific, catalog 88-5930-74). After the completion of staining, cells were resuspended in FACS buffer. Stained cells were run on a flow cytometer (BD LSRFortessa). Sample acquisition was done using FACSDiva software (BD) with subsequent analysis on FlowJo software.

To determine the presence of the ciliary protein ARL13b on human RBCs, unlabeled RBCs prior to infusion into the variable height flow chamber, and effluent containing RBCs collected postflow and RBCs postinteraction with brain ECs, were labeled with FITC-conjugated Arl13b antibody for 30 minutes on ice. RBCs were then washed and tested by flow cytometric analysis as previously described (49). To determine whether ROS generation in brain ECs can be increased by sickle RBCs and contributes to deciliation, slides coated with brain ECs were sham-treated or pretreated with the NOX inhibitor apocynin at 10 μM for 1 hour at 37°C, washed, and fitted into the flow chamber. Treated brain ECs were then exposed to unlabeled sickle RBCs for 10 minutes. Sickle RBCs were lysed with RBC lysis buffer and brain ECs scraped from the glass slide for testing for ROS levels using CM-H2-DCFDA (Invitrogen) as described previously in detail (50). One hundred thousand events per sample were acquired and tested by flow cytometric analysis. In separate experiments, and as described above, unlabeled sickle RBCs were tested by flow cytometry for ARL13b ciliary protein binding prior to infusion into the chamber (baseline levels) and postflow and once sickle RBCs interacted with brain ECs treated with 10 μM apocynin.

### Mouse sickle RBCs: cilia staining and quantification

For the blood smear preparation, approximately 5 μL of whole blood from AA control mouse and SS sickle mouse was added to a glass slide and allowed to dry for 24 hours. The smear was fixed with acetone for 10 minutes. At the end of incubation, the slides were washed with PBS, and the primary antibody (ARL13b or IFT88) at the concentration of 1:500 was added to the slides and incubated at 4°C overnight. The slides were washed with PBS, and secondary antibody was added at a concentration of 1:500 and incubated for 1 hour at room temperature in the dark. At the end of incubation, the slides were washed with PBS, the mounting reagent was added, and the coverslip was placed on top of the blood smear and imaged at 63× on a confocal microscope (Zeiss LSM 510 laser module) with bright-field for RBCs and 488 green channel for ARL13b- or IFT88-positive cilia detection. All the cilia-positive RBCs were quantified with multipoint tool in ImageJ software and represented as graphs.

### Statistics

Data were presented as mean and SEM. A 2-tailed *t* test or 1- or 2-way ANOVA was performed to compare groups on the outcomes. Pearson’s correlation and regression analysis were used to investigate the relationships between continuous variables. Cilia protein in vivo under different sheer stress was expressed as fold change relative to the mean of the control group. A linear mixed model (LMM) was then used to examine differences between experimental and control groups. The differences of cilia proteins (ARL13b, γ-tubulin, IFT88, and Inversin) within EC (mCherry^+^) or non-EC (mCherry^–^) were also analyzed by LMM. *P* < 0.05 was considered significant. Dunnett’s test, Tukey’s test, or Bonferroni correction was used to adjust for multiple comparisons. Data were log-transformed to improve fit for some analyses. Nonparametric tests were used where parametric assumptions were not satisfied. Statistical analysis was performed using SAS V9.4 (SAS Institute Inc.), R, and GraphPad Prism software (version 9.0).

### Study approval

#### Animal studies.

For this study, the zebrafish and mouse experiments at Medical College of Wisconsin were approved under the IACUC protocol 320 titled “Blood Vessel Development in Zebrafish” and 1022 titled “Molecular Genetics of Mouse Vascular Development and Associated Diseases.” Zebrafish studies at Qatar University were performed under approved AUA (QU-IBC-2020/074-REN1).

#### Human patient studies.

Collection of blood samples from human participants has been approved by Duke University’s Institutional Review Board (registry # Pro00007816), and written informed consent has been obtained from the participants.

## Author contributions

AG was responsible for designing research studies, conducting experiments, acquiring data, analyzing data, preparing figures, and writing the manuscript. KT was responsible for designing research studies, conducting experiments, acquiring data, preparing figures, and analyzing data. MT was responsible for designing research studies, conducting experiments, acquiring data, and analyzing data. AMM and HTZ were responsible for designing research studies, conducting experiments, acquiring data, preparing figures, and analyzing data. SP was responsible for designing research studies, conducting experiments, acquiring data, and analyzing data. MRG and ADS were responsible for designing research studies, conducting experiments, acquiring data, analyzing data, and editing the manuscript. AP was responsible for analyzing data, evaluating data, and preparing figures. SPP was responsible for intellectual input and editing the manuscript. HCY was responsible for designing research studies, analyzing data, preparing figures, and editing the manuscript. SMN was responsible for designing research studies, analyzing data, intellectual input, and writing and editing the manuscript. KRR and RZ were responsible for designing research studies, analyzing data, intellectual input, and writing and editing the manuscript. RR provided the conceptual framework, designed research studies, analyzed data, provided intellectual input, and wrote and edited the manuscript.

## Supplementary Material

Supplemental data

## Version 1 02/10/2022

In-Press Preview

## Version 2 03/22/2022

Electronic publication
