# Supplementary material for: Cilia proteins are biomarkers of altered flow in the vasculature
Source: JCI Insight. 2022 Mar 22;7(6):e151813. doi: 10.1172/jci.insight.151813 (PMC8986075; doi:10.1172/jci.insight.151813)
Supplement: Supplemental data [file jciinsight-7-151813-s035.pdf]

Cilia proteins are biomarkers of altered flow in the vasculature

Ankan Gupta[1,2,$], Karthikeyan Thirugnanam[1,2,$], Madhan Thamilarasan[3], Ashraf M. Mohieldin[4], Hadeel Tareq Mohammed Al-Jighefee[5], Shubhangi Prabhudesai[1,2], Meghan Griffin[1,6], Andrew D. Spearman[1,6], Amy Pan[1,7], Sean P. Palecek[8], Huseyin C. Yalcin[5], Surya M. Nauli[4], Kevin Rarick[9,$], Rahima Zennadi[3,$] and Ramani Ramchandran*[,1,2]

Supplemental figures

Fig. S1

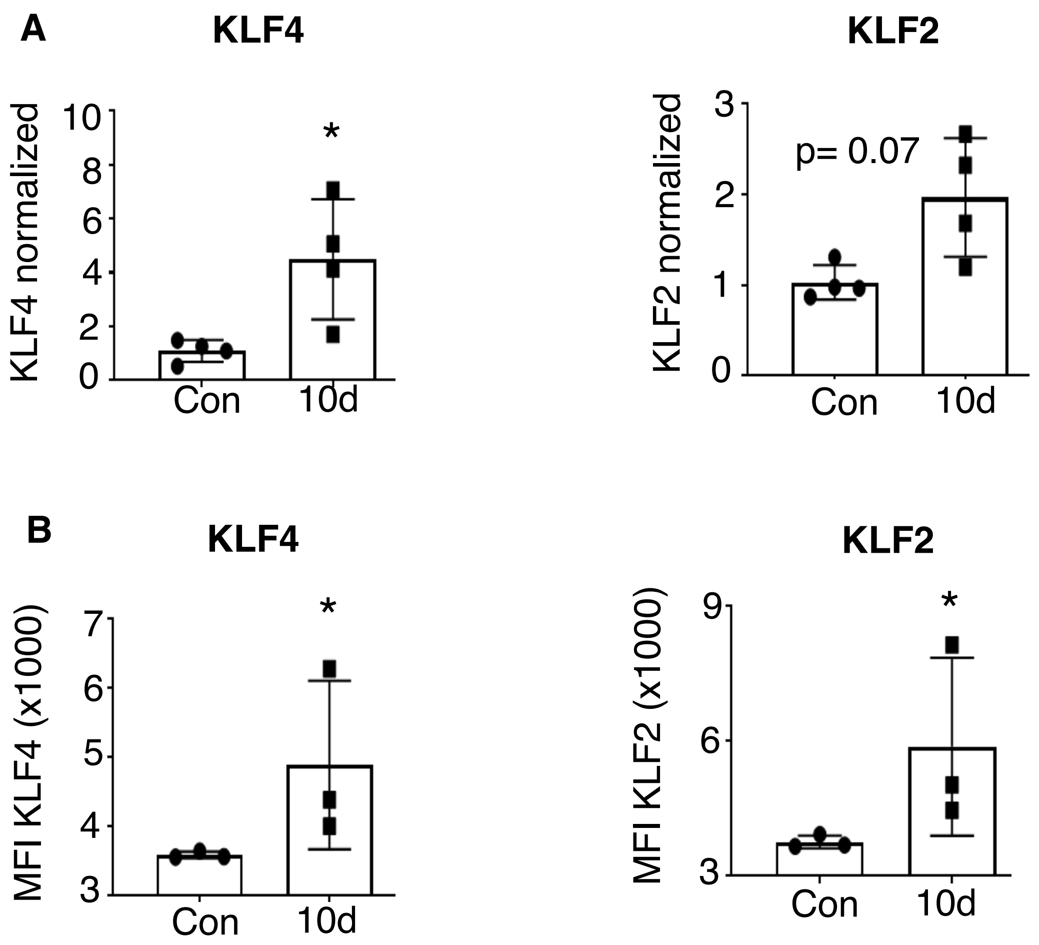

Figure S1: Validation of shear stress as induced by Ibidi flow system. Human brain microvascular endothelial cells were subjected to 10 dyne/cm^2 shear stress for 24 hours, as induced by ibidi flow system and subsequently monitored for flow responsive genes KLF4 or KLF2 by qRT-PCR (A) as well as the respective proteins by flow cytometry (B). KLF4 and KLF2 gene expressions were normalized against GAPDH and plotted as fold change. *P<0.05

Fig. S2

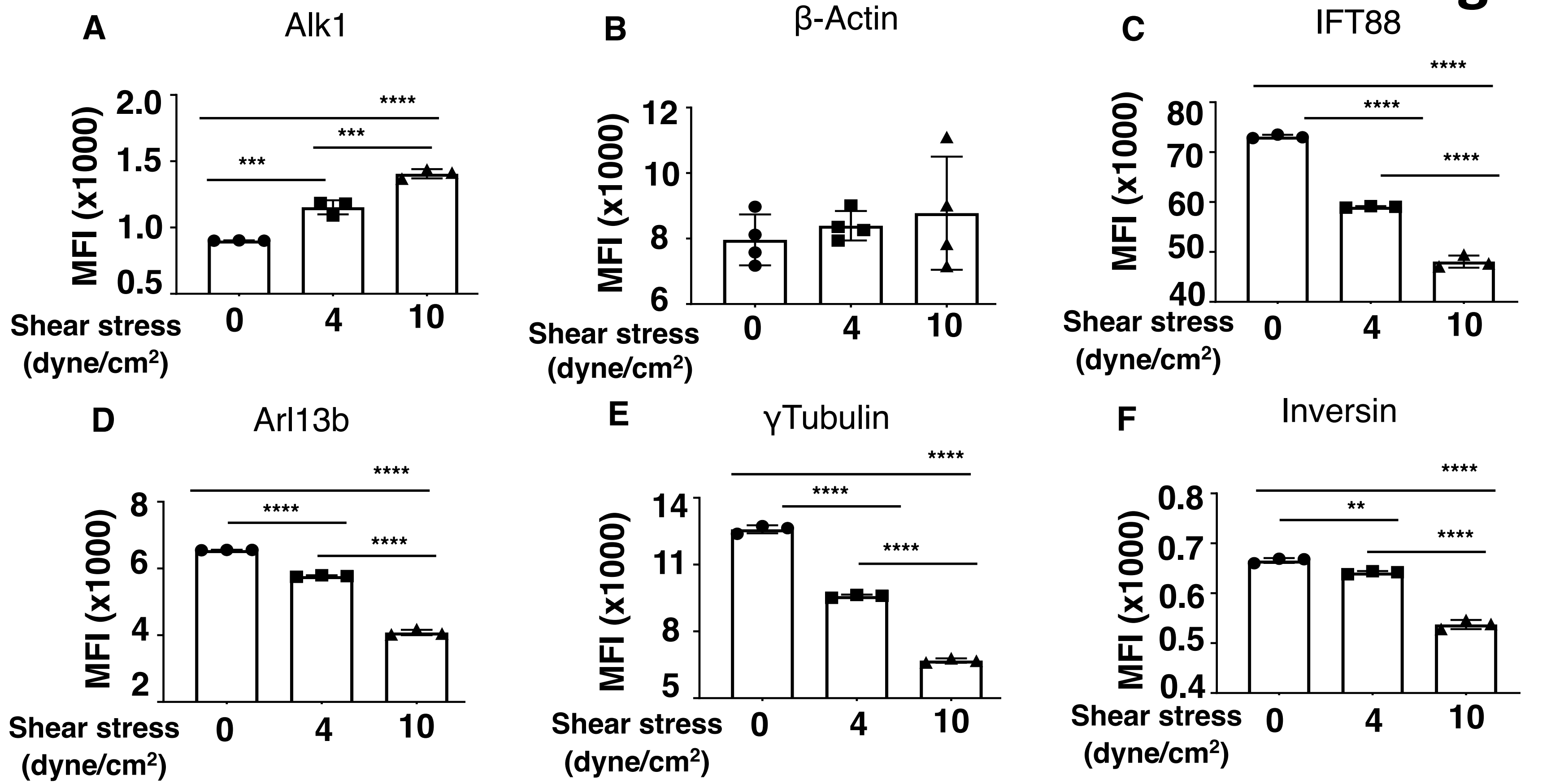

Figure S2: Loss of cilia proteins from brain endothelial cells following shear stress induced by shaker method *in vitro*. Human brain microvascular endothelial cells were subjected to graded strengths of shear stress (4 dyne/cm^2 and 10 dyne/cm^2) as induced by 'shaker' method and subsequently the expression of cilia-specific proteins was quantified by flow cytometry. Stress-responsive proteins were quantified (A) along with non-cilia house keeping (B) or cilia-specific proteins (C-F) by measuring median fluorescent intensity. Comparison across groups (4 dyne vs. Control, 10 dyne vs. Control and 4 dyne vs. 10 dyne) were performed for all proteins. In all three group comparisons, P<0.001 for Arl13b, Tubulin, IFT88, and Inversin proteins. For Alk1, P=0.0003 for 4 dyne vs. Control, P<0.001 for 10 dyne vs. Control and P=0.0004 for 4 dyne vs. 10 dyne comparison. For Dynein, P<0.001 for 4 dyne vs. Control and 10 dyne vs. Control, and P=0.0173 for 4 dyne vs. 10 dyne comparison. N=3 for all protein targets except for β-actin, which is n=4.

Fig. S3

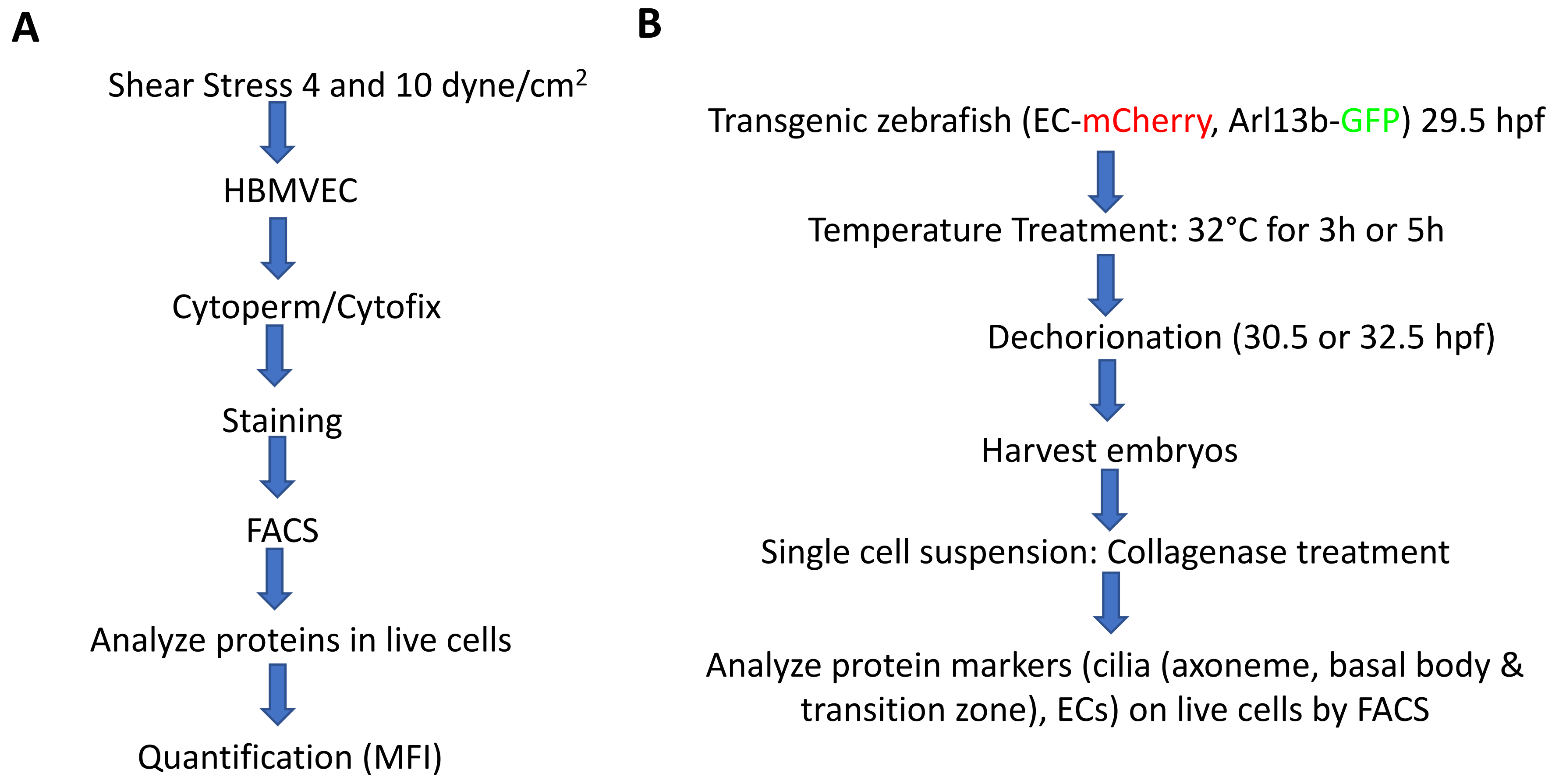

Figure S3: Scheme for shear stress EC FACS experiments *in vitro* and *in vivo*.
A shows the scheme the treatment of HBMVECs post shear stress for FACS analysis. B shows the scheme for fish treatment conditions, and subsequent analysis prior to FACS.

Fig. S4

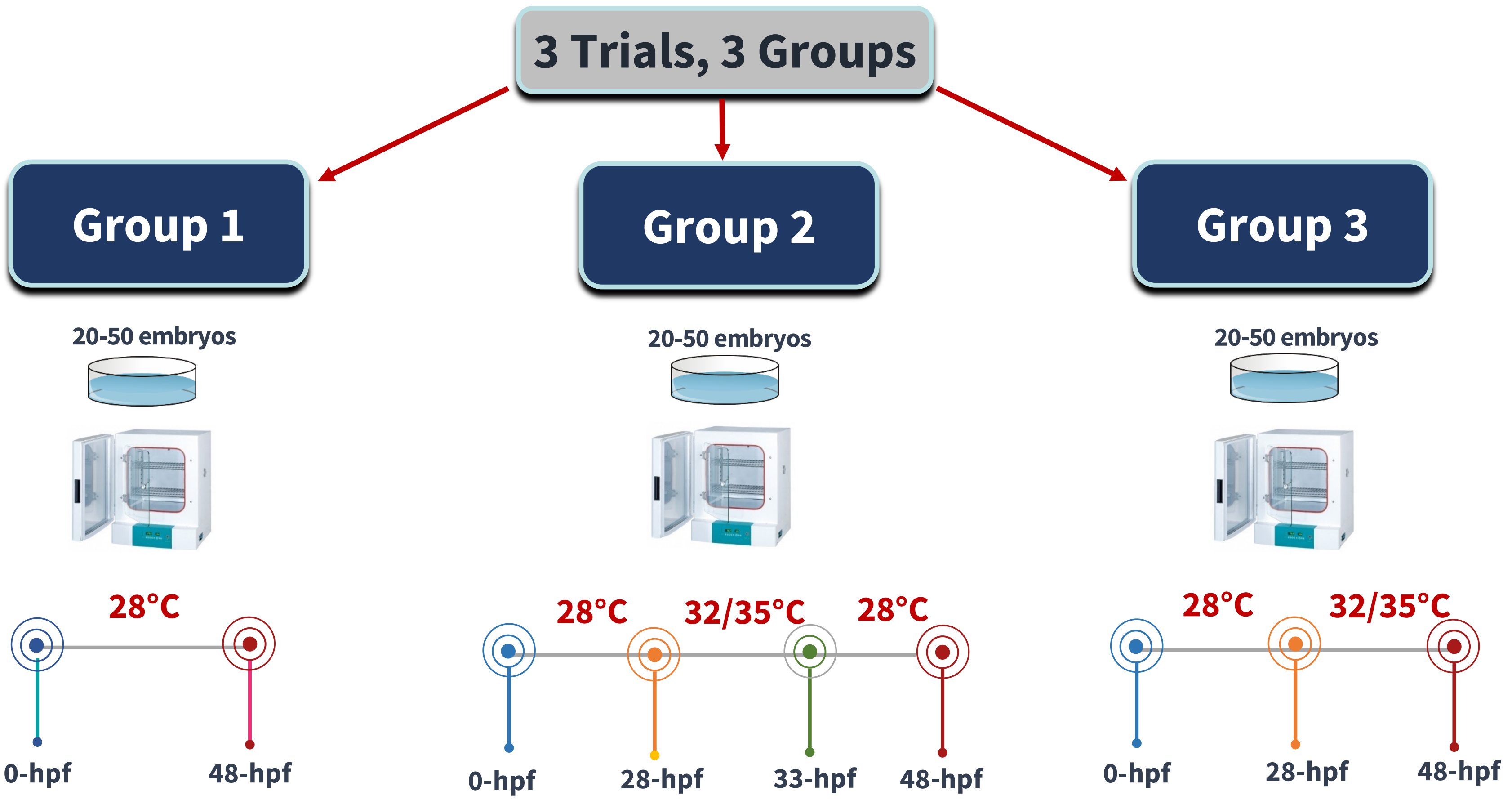

Figure S4: Temperature-induced shear stress experimental design in zebrafish.
The 3 groups and the conditions for incubation for each are depicted in a pictorial format. All blood flow parameters were evaluated at 48 hours post fertilization (hpf) stage.

Fig. S5

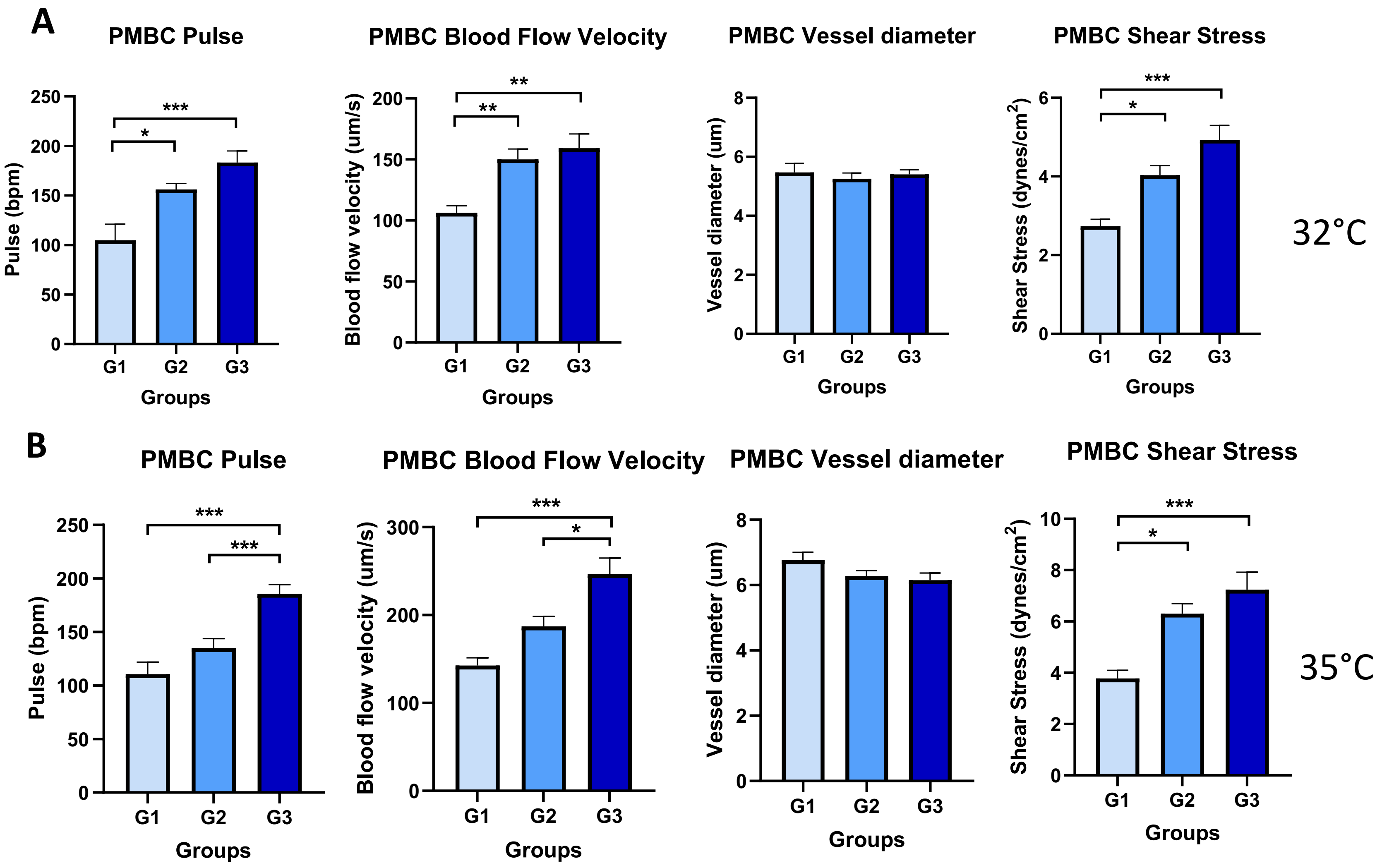

Figure S5: PMBC flow parameters assessed at two temperatures of 32°C and 35°C.
A shows pulse, blood flow velocity, vessel diameter and shear stress measured in primordial midbrain channels (PMBCs) across the three groups (G1-G3 shown in figure S2) at 32°C. B shows pulse, blood flow velocity, vessel diameter and shear stress measured in PMBCs across the three groups (G1-G3 shown in figure S2) at 35°C. *P<0.05, **P<0.01, ***P<0.001.

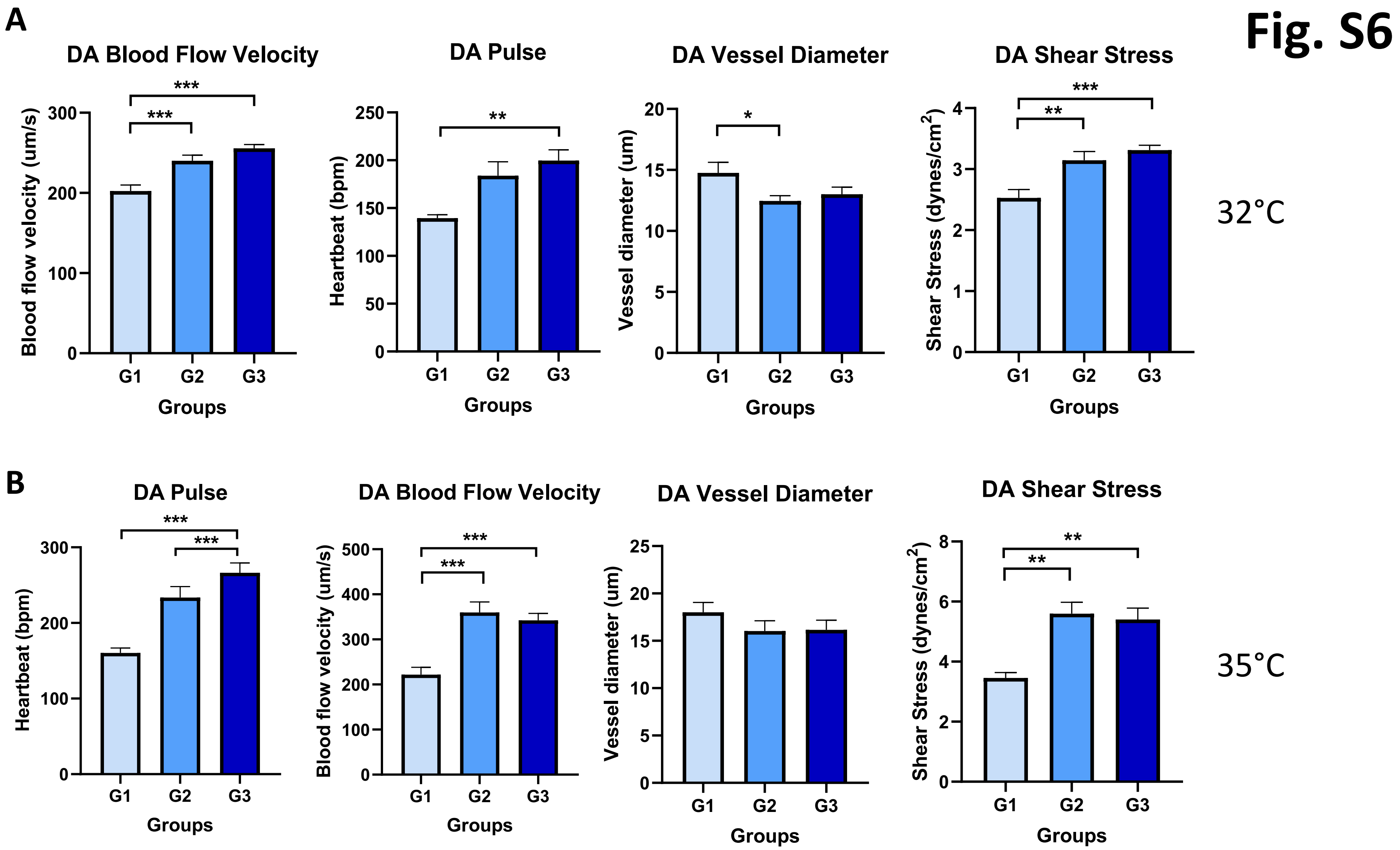

Figure S6: DA flow parameters assessed at two temperatures of 32°C and 35°C.
A shows pulse, blood flow velocity, vessel diameter and shear stress measured in dorsal aorta (DA) across the three groups (G1-G3 shown in figure S2) at 32°C. B shows pulse, blood flow velocity, vessel diameter and shear stress measured in DA across the three groups (G1-G3 shown in figure S2) at 35°C. *P<0.05, **P<0.01, ***P<0.001.

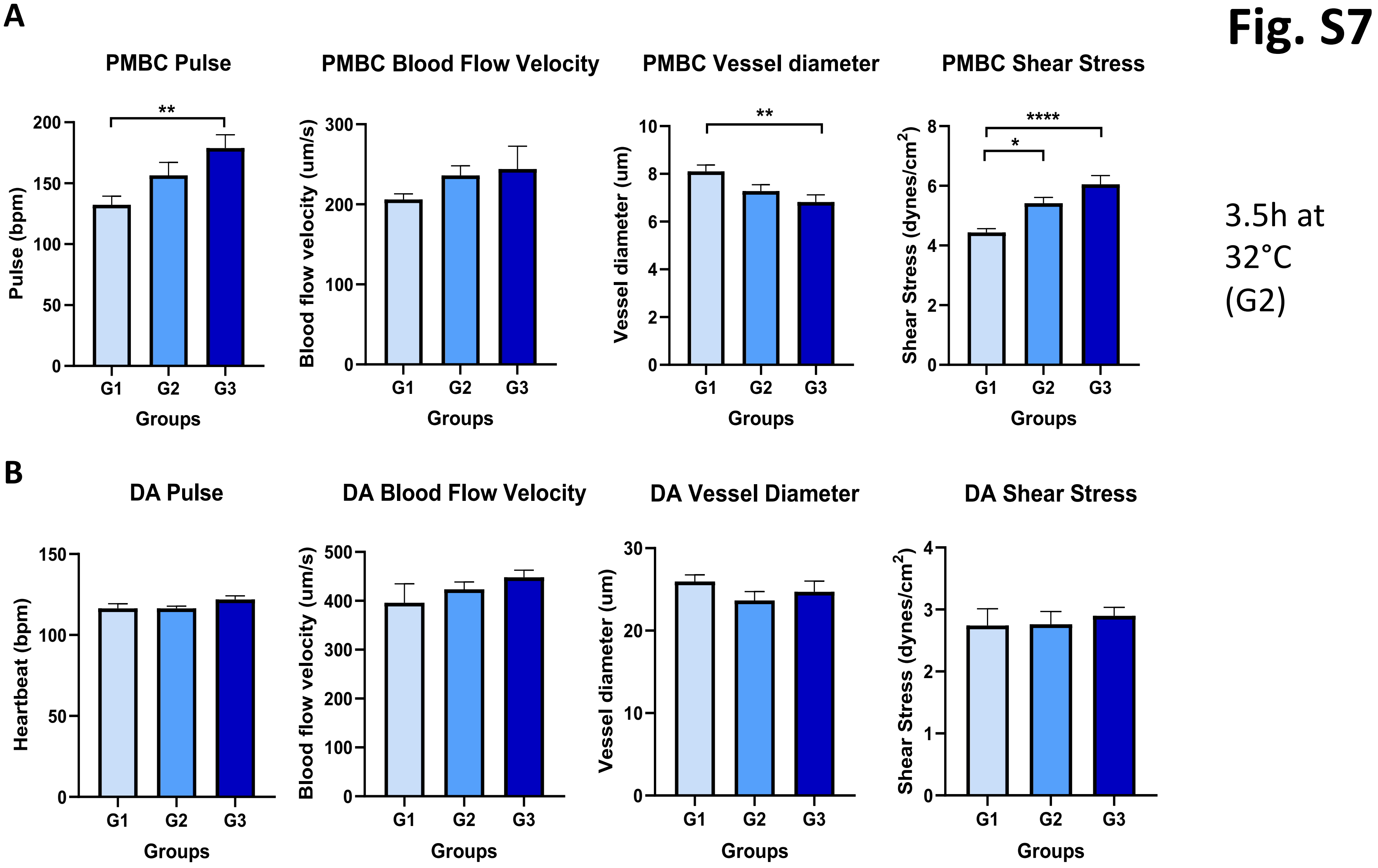

Figure S7: PMBCs and DA flow parameters assessed at two temperatures of 32°C.
A shows pulse, blood flow velocity, vessel diameter and shear stress measured in primordial midbrain channels (PMBCs) across the three groups (G1-G3 shown in figure S2) at 32°C. B shows pulse, blood flow velocity, vessel diameter and shear stress measured in DA across the three groups (G1-G3 shown in figure S2). *P<0.05, **P<0.01, ***P<0.001, ****P<0.0001.

Fig. S8

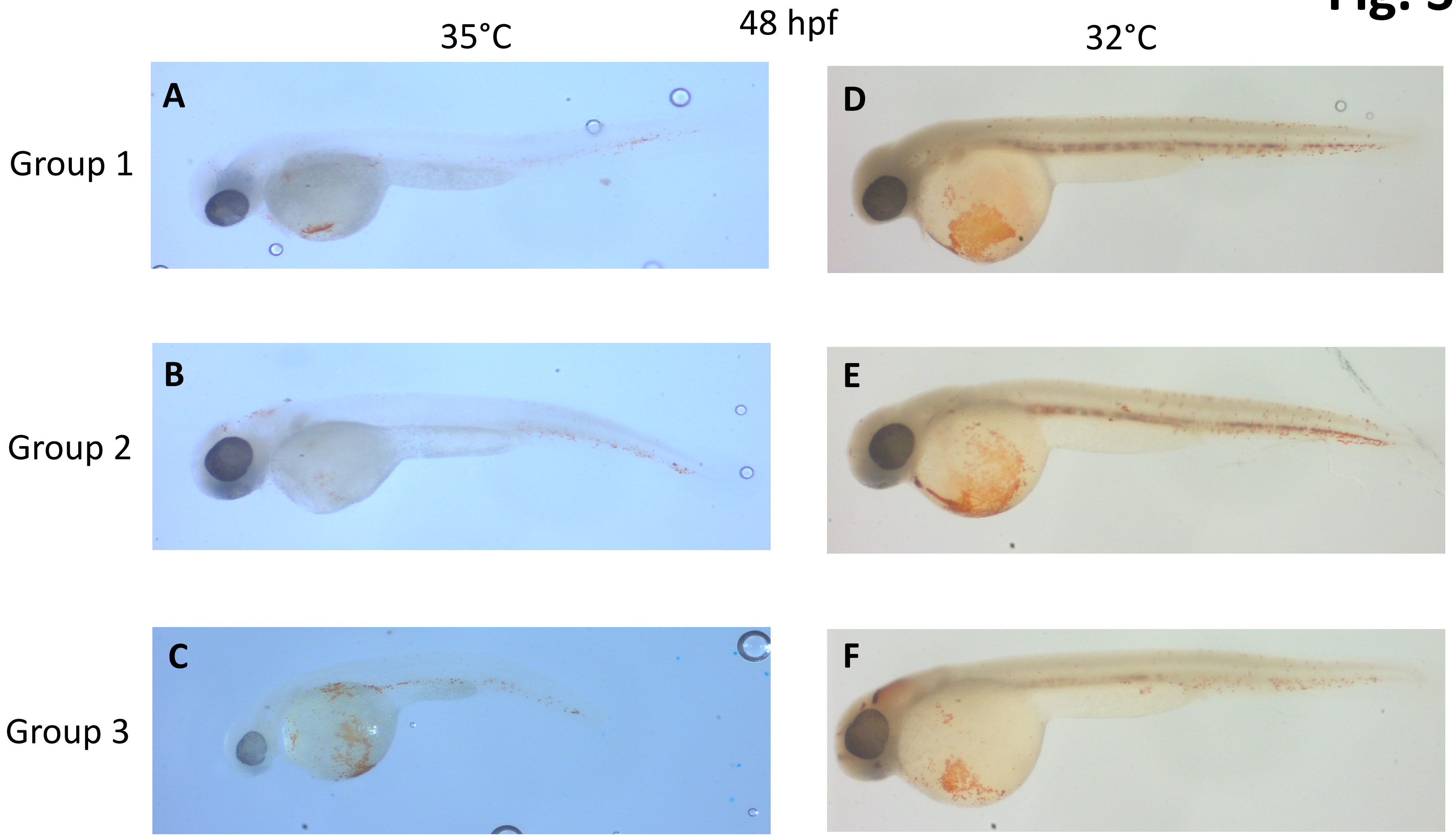

Figure S8: Whole mount images of casper fish at 35°C and 32°C.
A,D (group 1), B,E (group 2) and C,F (group 3) Casper transparent 48 hours post fertilization (48 hpf) fish embryos incubated at 35°C and 32°C were stained with O-dianisidine stain (red blood cells). Note group 3 (C) embryos at 35°C display curved axis. Anterior is left and posterior is right. Dorsal is up.

Fig. S9

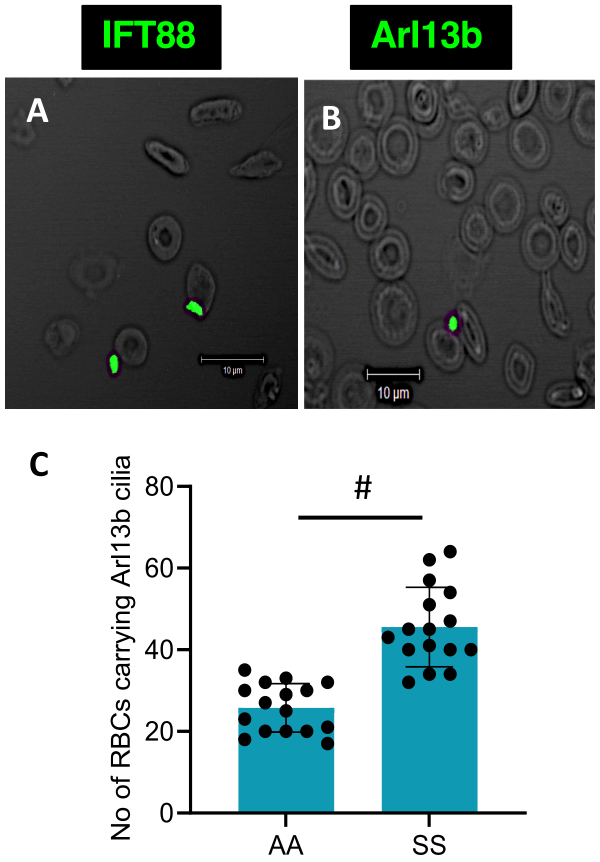

Figure S9. Cilia on circulating mouse sickle RBCs.
A and B shows blood smears from SS sickle mouse stained for IFT88 and Arl13b antibodies. The green stain is cilia on RBCs. Panel C quantification is from a different experiment from panels A & B. For the quantification of Arl13b positive cilia adhering to control (AA) vs. sickle RBCs (SS), four mice per group were used. 1 smear from each mice, and four fields in each smear were counted. Thus, a total of data from 16 smears counted from control mice and sickle mice group (n=4 per group) are presented. #P<0.0001.
